# Assessing Response to Therapy for Nontuberculous Mycobacterial Lung Disease: Quo Vadis?

**DOI:** 10.3389/fmicb.2018.02813

**Published:** 2018-11-20

**Authors:** Christopher Vinnard, Alyssa Mezochow, Hannah Oakland, Ross Klingsberg, John Hansen-Flaschen, Keith Hamilton

**Affiliations:** ^1^Public Health Research Institute, New Jersey Medical School, Newark, NJ, United States; ^2^Perelman School of Medicine, University of Pennsylvania, Philadelphia, PA, United States; ^3^Department of Medicine, Tulane University School of Medicine, New Orleans, LA, United States

**Keywords:** nontuberculous mycobactena, biomarker (development), response, therapeutics, clinical trial, radiography, quantitative culture

## Abstract

Assessing progression of disease or response to treatment remains a major challenge in the clinical management of nontuberculous mycobacterial (NTM) infections of the lungs. Serial assessments of validated measures of treatment response address whether the current therapeutic approach is on track toward clinical cure, which remains a fundamental question for clinicians and patients during the course of NTM disease treatment. The 2015 NTM Research Consortium Workshop, which included a patient advisory panel, identified treatment response biomarkers as a priority area for investigation. Limited progress in addressing this challenge also hampers drug development efforts. The Biomarker Qualification Program at the FDA supports the use of a validated treatment response biomarker across multiple drug development programs. Current approaches in clinical practice include microbiologic and radiographic monitoring, along with symptomatic and quality-of-life assessments. Blood-based monitoring, including assessments of humoral and cell-mediated NTM-driven immune responses, remain under investigation. Alignment of data collection schemes in prospective multicenter studies, including the support of biosample repositories, will support identification of treatment response biomarkers under standard-of-care and investigational therapeutic strategies. In this review, we outline the role of treatment monitoring biomarkers in both clinical practice and drug development frameworks.

Nontuberculous mycobacteria (NTM) are ubiquitous environmental organisms capable of causing significant morbidity (Yeung et al., 2016) and mortality (Vinnard et al., 2016; Diel et al., 2018), predominantly in the form of chronic lung disease (Wassilew et al., 2016). Profound knowledge gaps regarding the diagnosis of NTM lung infections (Griffith et al., 2007) include distinguishing NTM colonization from infection (van Ingen, 2015), identification of patients most likely to benefit from treatment (Mirsaeidi et al., 2014), selection of an optimal initial drug regimen (Philley and Griffith, 2015), and classification of treatment endpoints. As an explanation for these knowledge gaps, the formal study of NTM lung disease is relatively recent compared to tuberculosis, and basic understanding of NTM lung disease pathophysiology has only recently emerged.

In this review, we address uncertainties regarding the clinical “waypoints” of NTM treatment. Intermediate and definitive markers of treatment response provide crucial feedback to NTM patients, their clinicians, and developers of novel therapies. We outline the role of treatment monitoring biomarkers in both clinical practice and drug development. We summarize the existing knowledge base regarding biomarkers of treatment response for NTM disease and we identify areas for ongoing and potential future investigation.

## The Utility of a Treatment Response Biomarkers in Clinical Care and Drug Development

In his seminal framework for defining surrogate endpoints in clinical trials, Ross Prentice proposed that a valid surrogate endpoint must “capture” the underlying relationship between the intervention and the clinical endpoint, allowing for comparison of intervention groups based on the surrogate rather than the definitive clinical endpoint at any time during the follow-up period (Prentice, 1989). By comparison, the concept of a treatment monitoring biomarker is less restrictive than a surrogate endpoint. The Institute of Medicine defines a biomarker as “a characteristic that is objectively measured and evaluated as an indicator of normal biologic processes, pathogenic processes, or biologic responses to a therapeutic intervention” (Institute of Medicine (US) Committee on Qualification of Biomarkers and Surrogate Endpoints in Chronic Disease, 2010).

The BEST (Biomarkers, EndpointS, and other Tools) Resource, published by the FDA-NIH Biomarker Working Group, provides descriptions and examples of disease monitoring biomarkers, which are “assessed serially over time to measure presence, status, or extent of disease or medical condition, or to provide evidence of an intervention effect or exposure, including exposure to a medical product or environmental agent” (Cagney et al., 2017).

Thus, the interpretation of a monitoring biomarker is not based on a snapshot level, as with a baseline clinical predictor of response to therapy, but rather on changes in values during serial measurements. As examples, quantification of HIV RNA in blood serves as a monitoring biomarker for the clinical efficacy of antiretroviral therapy, and the amount of air that an individual can forcefully exhale in one second (FEV1) reflects response to chronic obstructive pulmonary disease management. Ideally, a treatment monitoring biomarker could be readily adopted into existing procedures that support routine use in clinical settings (Nahid et al., 2011).

The therapeutic approach to NTM lung disease lacks the evidence base of tuberculosis, where standard-of-care combination drug regimens are based on randomized clinical trials that enrolled tens of thousands of tuberculosis patients. Unlike tuberculosis, the NTM lung disease research agenda has not been propelled by public health pressure or urgency. Consequently, much of the clinical approach to NTM disease rests on a foundation of observational data and expert opinion, supported by only a few randomized clinical trials (Research Committee of the British Thoracic Society, 2001). In general, this evidence base has demonstrated that antimycobacterial therapies are less effective for NTM lung disease than tuberculosis (Griffith et al., 2007). The 2015 NTM Research Consortium Workshop, which included a patient advisory panel, identified treatment response biomarkers as a priority area for investigation (Henkle et al., 2016). One or more treatment response biomarkers would help to answer a fundamental question for clinicians and patients during the course of NTM disease treatment: is the current therapeutic approach on track toward a clinical cure?

This question has a particular significance for NTM disease given the complexity of treatment and the frequency of drug-related adverse events. Interval monitoring of treatment response would support clinical decision-making with regards to the intensification or de-escalation of therapies. When an initial period of intravenous therapy is used, for example with disease caused by *M. abscessus* complex, the optimal time point for transitioning to an all-oral regimen is uncertain. Surgical interventions may be the most appropriate approach for a subset of patients with severe *M. abscessus* complex lung disease, but potentially could be deferred in a subset of patients that are responsive to medical management (Jarand et al., 2011). Such decision nodes provide a potential point of application for a treatment monitoring biomarker. Monitoring biomarker response early in treatment could also inform clinical decision-making in advance of drug susceptibility testing (DST) results, which have a delay of several weeks and lack correlation between *in vitro* susceptibility and clinical outcomes for many antibiotics.

Aside from their value in clinical management decisions, treatment monitoring biomarkers support drug development efforts. According to guidelines established by the Food and Drug Administration (FDA), the use of a biomarker in the context of drug development relies on “qualification” that specifies its contextual use and interpretation. The Biomarker Qualification Program at the FDA provides a pathway for a particular biomarker to become accepted into the regulatory framework independent of approval of a specific drug (Amur et al., 2015). This program was recently updated in response to the 21st Century Cures Act, establishing formal qualification plans for biomarkers and other drug development tools. As an example, the Critical Path Institute (Tuscon, AZ, United States) is evaluating sputum levels of lipoarabinomannan, a component of the mycobacterial cell wall, as a non-culture based measure of tuberculosis treatment response, which could be incorporated into adaptive clinical trial designs (FDA, 2018a). Similarly, a qualified biomarker of treatment response for NTM disease would support drug development efforts across multiple programs, a notable advantage given the likelihood that combination antimicrobial therapies will remain essential to future drug regimens.

## Current Approaches to Monitoring Treatment Response to NTM Disease

### Microbiologic Monitoring

Presently, culture of serial sputum samples serves as the primary biomarker for monitoring the response to treatment of mycobacterial lung infections. In 2016, an expert panel composed of NTM clinicians reached consensus definitions for several endpoints related to the treatment of NTM lung disease, including cure, relapse, and re-infection (van Ingen et al., 2018). Serial culturing of sputum and deep respiratory samples during treatment of NTM lung disease provides the basis for definition of microbiologic cure, which is defined as 3 negative sputum cultures collected at least 4 weeks apart.

Interval microbiologic data may also provide insight into the effectiveness of therapy when the microbiologic cure endpoint has not yet been reached. Mycobacterial cultures from respiratory specimens can be classified semiquantitatively according to growth characteristics in liquid and solid media (Griffith et al., 2015; Table 1). Using this scoring system, Griffith et al. (2015) analyzed semiquantitative sputum culture scores on 180 patients with lung disease caused by *M. avium* complex (MAC), grouping patients into converters and non-converters based on sputum culture status after 12 months of treatment. Although baseline semi quantitative culture scores were not significantly different between converters and non-converters, the change in semi quantitative culture scores after 2 months of MAC-directed treatment was highly predictive of sputum culture conversion, with each 1-point decline in the scoring scale associated with a 20% increase in the likelihood of achieving conversion at 12 months. Importantly, early microbiologic response also correlated with symptomatic and radiographic improvements, suggesting that semi quantitative culture data can be explored as a surrogate endpoint in drug development. One limitation of this approach may be that some NTM lung disease patients do not expectorate sputum (Huang et al., 1999), although proper application of sputum induction technique considerably improves microbiologic yield (Guiot et al., 2017).

**Table 1:** A Semiquantitative Mycobacterial Culture Scoring System (Griffith et al., 2015).

Semi quantitative Culture Scores	Growth Scale	Growth in Broth	Growth on an Agar Plate	Countable Colonies on an Agar Plate
0	Negative	-	-	0
1	Positive broth only	+	-	0
2	1+	+	+	≤50
3	2+	+	+	50–99
4	2+	+	+	100–199
5	3+	+	+	200–299
6	4+	+	+	≥300

Interval treatment response monitoring based on microbiologic data is supported by recently published NTM lung disease treatment guidelines. The British Thoracic Society recommends obtaining interval sputum cultures every 4–12 weeks during treatment to assess microbiologic response (Haworth et al., 2017). Similarly, consensus recommendations provided by the US Cystic Fibrosis Foundation and the European Cystic Fibrosis Society for the management of NTM lung disease include interval monitoring of sputum culture every 4–8 weeks during treatment (Floto et al., 2016). An update from the American Thoracic Society/Infectious Disease Society of America regarding management of NTM lung disease is currently in preparation. Unlike tuberculosis, there are no data supporting non-culture based microbiologic monitoring during NTM lung disease treatment.

### Radiographic Monitoring

Radiographic imaging studies have long served an essential role, not only in diagnosing NTM lung infection and determining the extent of disease, but also in assessing response to treatment (Griffith et al., 2007). A simple chest radiograph scoring system with 3 tiers (improved, no change, or worsened in comparison with the preceding examination) was predictive of sputum culture conversion among MAC lung disease patients, with repeated routine chest radiography performed a mean of 59 days after the baseline (pre-treatment) examination (Griffith et al., 2015). Computed tomographic (CT) imaging considerably improves assessment and comparison of NTM disease extent compared to chest radiographs. Indeed, several scoring criteria for CT imaging studies have been developed and evaluated for this purpose (Kim et al., 2012; Lee et al., 2013).

Untreated patients with NTM lung disease demonstrate progression of CT findings on serial studies that inform decisions to initiate treatment, particularly with the nodular bronchiectatic form of MAC lung disease (Park et al., 2017). Similar to microbiologic endpoints, radiographic improvement at the conclusion of therapy can characterize successful treatment of NTM lung disease. However, less is known regarding the contribution of early radiographic changes as a marker of treatment response. Moreover, interval radiographic findings during treatment of NTM lung disease may be confounded by the extent of underlying lung disease and by the presence of concomitant infection or colonization of airways (Park et al., 2016; Cohen-Cymberknoh et al., 2017). Other limitations of serial CT imaging for monitoring treatment response include the difficulty with classification of NTM lung disease patients with fibrocavitary disease (an alternate form of MAC lung disease), or structural lung changes due to additional treatment modalities (such as surgical resection or radiation fibrosis) (Lee et al., 2013).

**FIGURE 1:**
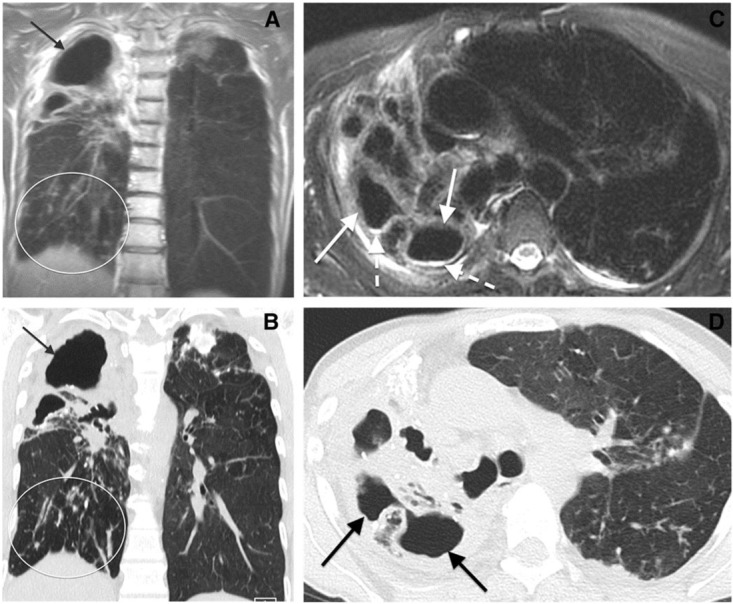
Comparison of chest MR to CT imaging for assessment of *Mycobacterium avium* complex lung infection. Cavitary Mycobacterium avium complex (MAC) pneumonia in a 64-year-old woman. **(A)** Coronal MR and **(B)** coronal computed tomographic (CT) images show cavitary disease (arrows) and traction bronchiectasis in the right upper lobe. Subcentimeter nodules (oval) are present in the right lower lobe. **(C)** Axial MR and **(D)** CT images of the upper lungs show cavitary disease (solid arrows) and traction bronchiectasis in the right upper lobe. Reproduced with permission from Chung et al. (2016).

Patients with active chronic lung diseases are exposed to significant radiation when monitored by chest CT imaging. Because proton magnetic resonance imaging (MRI) does not use ionizing radiation this imaging modality has been studied for assessment of cystic fibrosis and sarcoidosis, performing comparably to chest CT in identifying various pathologic patterns of the disease in lung parenchyma. Chung and associates have proposed that MRI may emerge as a viable alternative to CT imaging for assessing the distribution and extent of NTM lung infection (Chung et al., 2016). In an exploratory study of 25 patients known to have lung infection with MAC, they found excellent agreement between noncontrast MRI and CT imaging for detection of nodules and cavities, but only limited agreement for detection of bronchial mucous plugging (Chung et al., 2016; Figure 1). The authors postulated that further improvement in special discrimination of NTM lung infection may be achievable by application of maximum intensity projection reconstruction and contrast enhancement. To our knowledge, no published studies have reported on the usefulness of MRI for serial assessment of NTM lung infection.

**FIGURE 2:**
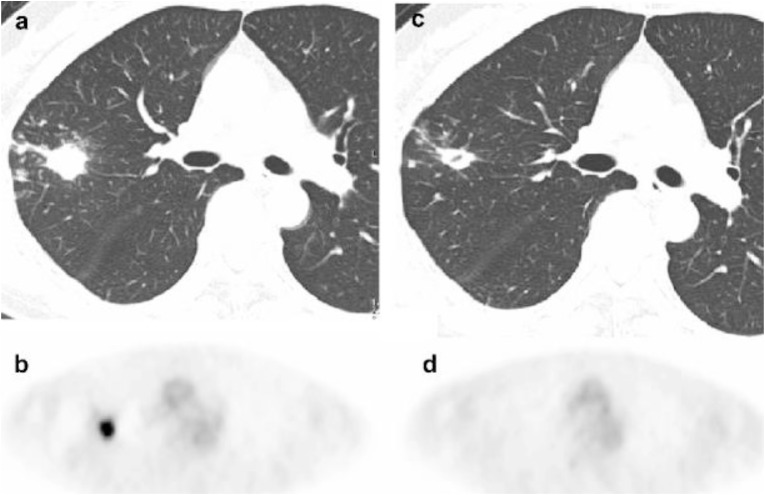
^18^F-FDG PET imaging before and after treatment of mycobacterium avium complex lung infection. Thoracic images obtained from a patient with mycobacterium avium complex lung infection before and after treatment. **(a)** An axial CT image shows an untreated nodule **(b)** ^18^F-FDG PET image reveals intense accumulation in the nodule (SUV 7.5) in the untreated nodule. **(c)** A CT image shows persistence of the nodule with interim cavitation after treatment. **(d)** ^18^F-FDG PET image shows complete absence of FDG accumulation in the nodule after treatment. Reproduced with permission from Demura et al. (2009).

Interval examination with ^18^F-fluorodeoxyglucose positron-emission tomography [^18^F-FDG PET] is a routine component of monitoring response to chemotherapy for lung cancer, based on the detection of increased glucose metabolism that is characteristic of malignant lesions. More recently, the use of PET modalities has been examined for diagnosis and treatment monitoring of tuberculosis, detecting the increase in glucose metabolism associated with the host inflammatory response (Vorster et al., 2014). Nodular MAC lung disease may show a similar pattern of increased glucose metabolism on PET/CT imaging, raising the possibility of interval monitoring during treatment. Among 6 patients with MAC lung disease assessed with ^18^F-FDG PET studies before and after MAC-directed treatment, all patients demonstrated a decline in ^18^F-FDG uptake after treatment, even in the absence of significant improvement in high-resolution CT images (Demura et al., 2009; Figure 2).

### Symptomatic Improvement and Quality-of-Life Monitoring

The concept of clinical cure of NTM disease is tied to symptomatic improvement at the conclusion of treatment, which may not reach complete resolution of symptoms due to underlying structural lung disease (van Ingen et al., 2018). A number of studies have measured baseline quality-of-life indicators among NTM lung disease patients using various assessment tools, including the St. George Respiratory Questionnaire (SGRQ) (Mehta and Marras, 2011), the EuroQOL Five Dimensions (Hong et al., 2014), and the Medical Outcomes Short-Form-36 Questionnaire (SF-36) (Mehta and Marras, 2011; Asakura et al., 2015). However, there are a paucity of data regarding the formal assessment of quality-of-life during NTM treatment as an interval marker of clinical response, a notable knowledge deficit given the importance of patient reported outcomes in the context of drug development efforts. Importantly, under the 21st Century Cures Act, the FDA has updated the regulatory framework for evaluating these types of patient-reported outcomes in the Clinical Outcome Assessment Qualification Program, which operates in parallel with the Biomarker Qualification Program to support the evaluation of drug development tools for a disease area (FDA, 2018b).

Czaja et al. (2016) studied 47 patients with MAC lung disease, assessing quality-of-life at regular intervals during treatment with the SGRQ, and observing a significant improvement as early as 3 months from the treatment initiation. Notably, improvements in quality-of-life assessments were not related to improvements in CT imaging, perhaps a reflection of the limitations of CT imaging as discussed above, along with the greater degree of overall disease status and complexity that can be captured by quality-of-life assessments. A cross-sectional study of MAC complex lung disease patients, based on the SF-36, found that quality-of-life scores were lower among currently treated patients compared to previously- or never-treated patients, but repeated assessments during treatment were not performed (Asakura et al., 2015). While one interpretation of these findings would be that current MAC treatment modalities negatively affect quality-of-life, an alternate explanation would be that patients are selected for MAC treatment based on current symptoms, as reflected in quality-of-life scores.

### Blood-Based Monitoring

Both humoral and cell-mediated NTM-driven immune responses have been examined as potential sources of treatment monitoring biomarkers. As an alternative to sputum-based biologic sampling, blood-based monitoring may offer several advantages. Unlike sputum, blood is readily available throughout the treatment course, and continuously distributed biomarkers in blood would support precise comparisons with baseline levels without relying on hierarchical scoring systems (as used for radiographic findings or microbiology results). Furthermore, patients undergoing treatment for extra-pulmonary forms of NTM disease could potentially benefit from a validated blood-based biomarker of treatment response, for example during treatment of deep tissue infections such as osteomyelitis, where clinical response is less apparent on physical examination. Immune-based biomarkers of treatment response may also shed insight into the immunologic correlates of protection against disease in at-risk patient populations.

The Capilia MAC Ab ELISA kit (TAUNS, Shizuoka, Japan) developed for the diagnosis of MAC lung disease, measures serum levels of IgA antibodies against glycopeptidolipid (GPL), a major component of the MAC cell wall (Shibata et al., 2016). Serum anti-GPL IgA levels have been proposed as potential biomarkers of treatment response (Jhun et al., 2017; Kitada et al., 2017), although clinical evidence is limited to studies where serum anti-GPL IgA levels were compared before and after treatment, rather than at earlier timepoints during treatment.

Kitada et al. (2005) followed 27 patients with pulmonary MAC disease, obtaining serum samples before and after anti-MAC therapy. Among patients achieving clinical cure, a reduction in anti-GPL IgA levels was observed, while no difference was observed among 13 patients with treatment failure. Interestingly, a single patient with medical treatment failure then underwent surgical lobectomy, and subsequently demonstrated a fall in serum anti-GPL IgA levels, corresponding to conversion of sputum cultures. In a subsequent study that included 34 patients with MAC lung disease, serum anti-GPL IgA post-treatment declined from pre-treatment values among 19 patients who achieved sputum culture conversion, but not among 7 patients who either had disease recurrence or 8 patients who failed to achieve conversion (Kitada et al., 2017).

Jhun et al. (2017) evaluated serial changes in serum anti-GPL IgA levels during treatment of MAC lung disease at baseline, 3 months, and 6 months of treatment. Across all 57 patients, a decrease in serum anti-GPL IgA levels was observed. In contrast to the earlier findings discussed above (Czaja et al., 2016), patients with unfavorable treatment responses also had significant declines in anti-GPL IgA levels, although baseline levels were higher among patients who eventually developed treatment failure, perhaps reflecting a greater initial disease burden.

Cell-mediated immune responses, rather than antibody responses, could provide an alternate approach for immunologic monitoring of treatment response. Kim et al. (2014) enrolled 42 patients with MAC lung disease and measured serum concentrations of 36 different type I cytokine-associated molecules before and after 12 months of treatment. Declining levels of IL-17 and IL-23 during follow-up were associated with treatment failure, suggesting that continued impairment of the IL-17 pathway reflects ongoing disease processes. These findings are consistent with earlier work demonstrating that low IL-17 levels are a risk factor for developing MAC lung disease (Lim et al., 2010). Depending on the temporal dynamics of serum cytokines in the IL-17 pathway earlier in treatment, the measurement of one or more molecules on this pathway could be of interest as a potential monitoring biomarker.

Carbohydrate antigen 19-9 (CA 19-9) is a treatment monitoring biomarker for several forms of cancer. Elevated blood levels of CA 19-9 are also seen in various forms of chronic lung disease, including bronchiectasis. Recently, CA 19-9 was examined as biomarker of treatment response among 24 patients with NTM lung disease in South Korea (Hong et al., 2016). Among 17 patients with treatment response (defined as symptomatic improvement and sputum culture conversion within 12 months of therapy), CA 19-9 levels significant declined, while no difference was observed among the 7 patients without clinical response. Larger cohort studies with serial measurements of CA 19-9 earlier in treatment may be of interest.

### Pulmonary Function Monitoring

Measurement of forced expiratory volume is a prognostic indicator for several chronic respiratory diseases, including COPD and cystic fibrosis, and the FEV1 is a qualified biomarker for COPD drug development efforts. The utility of pulmonary function monitoring as a general biomarker of treatment response for NTM lung disease will likely be limited by the tremendous heterogeneity in underlying chronic lung diseases and NTM disease presentations (for example, nodular bronchiectatic vs. fibrocavitary disease). In a single center study of 37 cystic fibrosis patients with pulmonary *M. abscessus* infection, early improvements in FEV1 after treatment initiation (either 30 or 60 days) were not associated with long-term microbiologic outcomes or sustained improvements in pulmonary function (DaCosta et al., 2017).

## Future Directions

Clinical research centers specializing in the treatment of NTM lung disease will benefit from alignment of prospective data collection schemes that include specimen repositories for blood (both plasma and peripheral blood mononuclear cells), sputum, and urine. The Bronchiectasis and NTM Research Registry, originally designed to enroll patients with bronchiectasis without underlying cystic fibrosis, supports serial banking of blood and deep respiratory specimens (Aksamit et al., 2017). This framework could provide the basis for investigating some of the potential microbiologic and serum and biomarkers discussed above. Future advances in MRI and PET imaging and in quantitative comparison of serial imaging studies may accelerate future research on changes in anatomic extent and metabolic activity of NTM infections in response to treatment.

Complementary prospective, observational studies would support comprehensive phenotyping of NTM disease patients with serial assessments of novel biomarkers of disease status and treatment response, and potentially could compare treatment monitoring biomarkers between patients treated for pulmonary and extra-pulmonary NTM disease. Prospective studies of novel monitoring biomarkers should also consider the potential for ongoing environmental re-exposure to NTM pathogens leading to re-infection, as can occur with contaminated household water sources (Lande et al., 2018) or nosocomial infections (Baker et al., 2017). Finally, much of the treatment response work thus far has focused on NTM lung disease has centered on MAC, and multicenter enrollment strategies may be essential to study response biomarkers for non-MAC lung disease, in particular lung disease caused by *M. abscessus* complex. These types of large scale, multicenter studies will also be essential to the prospective evaluation of novel treatment regimens for NTM lung disease. Multiple enrollment sites will be required to provide the statistical power for comparing alternative therapeutic strategies, given the smaller number of NTM lung disease patients compared with tuberculosis on a global scale. Furthermore, clinical studies supported by the National Institutes of Health (Sigal et al., 2017) and the Bill & Melinda Gates Foundation (Chen et al., 2017) have demonstrated the feasibility of integrating biomarker assessment into clinical trial design.

## Summary

“Quo vadis” (“whither goest thou?”) remains a central question for NTM patients and their physicians. It is uncertain whether a single biomarker of clinical response will sufficiently capture the complexity of NTM disease presentations in a heterogeneous patient population, or whether the current “all of the above” strategy can be improved with refinement of microbiologic, radiographic, and quality-of-life assessments. Should a composite biomarker be developed under the FDA Biomarker Qualification program, it would require a “Context of Use” defining the specific algorithm for combining these individual components into a single treatment response measure. Ongoing research efforts for NTM disease should support the evaluation of biomarkers of response to treatment in parallel with studies of novel therapeutics and mechanisms of disease. Patients, clinicians, and investigators will benefit from the knowledge that the road they travel leads toward clinical cure.

## Author Contributions

AM, CV, JH-F, and KH performed the literature review. CV wrote the initial draft. AM, HO, RK, JH-F, and KH critically appraised and revised.

## Conflict of Interest Statement

The authors declare that the research was conducted in the absence of any commercial or financial relationships that could be construed as a potential conflict of interest.
